# Stroke-associated pneumonia in Japanese acute care settings: incidence and preliminary validation of risk prediction scores

**DOI:** 10.1186/s12883-025-04523-8

**Published:** 2025-12-11

**Authors:** Hiroki Kimura, Rika Hasegawa, Hiromi Oku

**Affiliations:** 1 Brain Attack Center Ota Memorial Hospital, Hiroshima, Japan; 2 Graduate School of Nursing, St. Luke’s International University, Tokyo, Japan

**Keywords:** Stroke-associated pneumonia, Risk prediction scores, A^2^DS^2^, Acute stroke care, Japan, Dysphagia

## Abstract

**Background:**

Stroke-associated pneumonia (SAP) significantly impacts mortality and functional outcomes in acute stroke care. While multiple risk prediction scores have been developed internationally, their performance in Japanese healthcare settings—characterized by high nurse-to-patient ratios and systematic multidisciplinary protocols—remains unexplored. This study aimed to determine SAP incidence in Japanese acute stroke care and validate established risk prediction scores.

**Methods:**

We conducted a retrospective cohort study of consecutive patients with first-ever acute stroke admitted within 7 days of onset to a 199-bed acute care hospital with a dedicated 24-bed stroke care unit (April 2022–March 2023). SAP was diagnosed using modified CDC criteria requiring radiographic evidence. Three validated risk scores (A^2^DS^2^, Pneumonia score, ISAN) were calculated and compared using receiver operating characteristic analysis. Independent predictors were identified through multivariable logistic regression using directed acyclic graph-based variable selection.

**Results:**

Among 847 patients analyzed (median age 75 years, 54.2% male), SAP developed in 22 patients (2.6%, 95% CI 1.5–3.7). Most cases (77.3%) occurred within 72 h. All three scores demonstrated good discrimination: A^2^DS^2^ achieved the highest AUROC of 0.825 (95% CI 0.78–0.88), followed by Pneumonia score and ISAN (both AUROC 0.798). No statistically significant differences were observed between scores. An A^2^DS^2^ cutoff ≥ 6 provided optimal balance (sensitivity 86.4%, specificity 65.8%, NPV 99.5%). Independent predictors included male sex (OR 3.87, 95% CI 1.41–10.61), NIHSS score (OR 1.08 per point, 95% CI 1.03–1.12), dysphagia (OR 5.31, 95% CI 1.16–24.34), and mechanical ventilation (OR 5.33, 95% CI 1.28–22.18).

**Conclusions:**

SAP incidence in Japanese acute stroke care (2.6%) was substantially lower than international reports, likely reflecting high nursing standards and systematic preventive protocols. Despite low baseline risk, the A^2^DS^2^ score demonstrated excellent discrimination and high negative predictive value, enabling reliable risk stratification. The predominance of early SAP onset supports intensive monitoring during the critical first 72 h. These findings support implementation of A^2^DS^2^-based risk stratification protocols in Japanese stroke care settings.

**Supplementary Information:**

The online version contains supplementary material available at 10.1186/s12883-025-04523-8.

## Introduction

Stroke-associated pneumonia (SAP) is defined as a lower respiratory tract infection occurring within 7 days after stroke onset, with reported incidence rates varying widely from 2.3% to 44% [1, 2]. SAP significantly impacts mortality, functional outcomes, and healthcare costs [3].

The variation in SAP incidence may be attributable to differences in diagnostic definitions and patient characteristics, as well as regional differences in healthcare delivery systems. Sari et al. (2017) reported differences in SAP incidence between Stroke Care Unit (SCU) in Indonesia and Japan, suggesting that healthcare system variations may influence SAP epidemiology [4].

To identify patients at high risk for SAP, several risk prediction scores have been developed, including the A^2^DS^2^ score (Age, Atrial fibrillation, Dysphagia, Sex, stroke Severity; National Institutes of Health Stroke Scale [NIHSS]), the Acute Ischemic Stroke-Associated Pneumonia Score (AIS-APS), the ISAN score (prestroke Independence, Sex, Age, NIHSS), and the Pneumonia score [5–9]. While recent meta-analyses comparing these risk prediction scores have suggested that AIS-APS may be superior, other reports indicate no significant performance differences between scores [10–12].

The Japanese healthcare system possesses unique characteristics that may influence SAP epidemiology. These include nurse staffing standards based on the reimbursement system, the presence of primary stroke centers certified by the Japan Stroke Society, multidisciplinary care involving speech-language pathologists and dental hygienists, and implementation of swallowing screening protocols. However, SAP epidemiology and risk score performance in the Japanese population have not yet been explored.

Therefore, this study selected three scores—A^2^DS^2^, Pneumonia score, and ISAN—based on the following criteria: (1) consisting solely of clinical variables readily available at admission, (2) having undergone extensive external validation in European or Asian populations, and (3) demonstrating clinical utility across diverse healthcare settings. We aimed to evaluate their discriminative ability and calibration in the Japanese healthcare environment.

## Methods

### Study design and participants

This retrospective cohort study was conducted at Ota Memorial Hospital, a 199-bed acute care facility with a dedicated 24-bed SCU, from April 1, 2022, to March 31, 2023. All consecutive patients aged 18 years or older admitted within 7 days of symptom onset for first-ever acute stroke (ischemic stroke, intracerebral hemorrhage, subarachnoid hemorrhage) were included.

Exclusion criteria were: (1) history of stroke, (2) death or transfer within 24 h of admission, (3) admission beyond 7 days after stroke onset, (4) in-hospital stroke, (5) age under 18 years, and (6) incomplete data for primary outcome assessment.

### Sample size calculation

Sample sizes were calculated for multiple objectives. For incidence estimation with an expected SAP rate of 10% (95% CI ± 3), 385 cases were required. For ROC (Receiver Operating Characteristic) analysis with expected AUC (Area Under the Curve) 0.70–0.75, significance level 0.05, and power 80–90%, the Hanley & McNeil method indicated 800–1000 cases were needed. For multivariable logistic regression with an expected 5-variable model, assuming 10 events per variable, a minimum of 50 SAP events was estimated. Considering the actual event rate of 2.6%, the final sample provided sufficient power for ROC analysis but limited power for multivariable modeling (events per variable = 4.4).

### Minimization of potential bias

The following strategies were implemented to minimize potential bias:

#### Selection bias

All consecutive patients meeting eligibility criteria during the study period were included without selection.

#### Information bias

Pre-defined diagnostic criteria based on modified CDC (Centers for Disease Control and Prevention) guidelines were consistently applied. All outcome assessments were based on objective clinical and radiological findings recorded in medical records.

#### Confounding

A Directed Acyclic Graph (DAG) approach was used to identify the minimal adjustment set for confounding control, ensuring appropriate variable selection for multivariable analysis.

### Data collection

Clinical variables were systematically extracted from electronic medical records by trained research staff using standardized data collection forms. Variables included age, sex, stroke type, pre-stroke modified Rankin Scale (mRS) [13], admission NIHSS, hypertension, hyperlipidemia, diabetes mellitus, atrial fibrillation, alcohol history, smoking history, enteral feeding, mechanical ventilation, and dysphagia.

Dysphagia assessment was performed by speech-language pathologists. The Fujishima Dysphagia Grade [14] or Modified Mann Assessment of Swallowing Ability (MASA) [15] was applied according to the patient’s consciousness level and general condition. Dysphagia was defined as MASA score < 178 or Fujishima Dysphagia Grade ≤ 7.

### SAP determination

SAP was determined according to modified CDC criteria proposed by the Pneumonia in Stroke Consensus Group [1]. Diagnosis required all of the following criteria to be met within 7 days of stroke onset:

At least one of the following systemic symptoms:

1. Fever (> 38 °C) with no other recognized cause.
2. Leukopenia (< 4,000 WBC/mm³) or leukocytosis (> 12,000 WBC/mm³).
3. For adults ≥ 70 years old, altered mental status with no other recognized cause.

At least two of the following:

1. New onset of purulent sputum, or change in character of sputum over a 24-hour period, or increased respiratory secretions, or increased suctioning requirements.
2. New onset or worsening cough, or dyspnea, or tachypnea (respiratory rate > 25/min).
3. Rales, crackles, or bronchial breath sounds.
4. Worsening gas exchange (e.g., oxygen desaturation [e.g., PaO₂/FiO₂ ≤240], increased oxygen requirements)

And ≥ 2 serial chest radiographs with at least 1 of the following:

New or progressive and persistent infiltrate, consolidation, or cavitation Note: In patients without underlying pulmonary or cardiac disease, 1 definitive chest radiograph is acceptable.

Chest X-ray findings were determined based on attending physician clinical records or official radiologist reports recorded in medical records. Diagnosis by chest CT was also included under the same criteria. Only confirmed SAP cases meeting all criteria were included, and suspected cases lacking radiographic diagnosis were excluded from primary analysis.

### Risk score calculation

Three validated risk scores were calculated for each patient:

A^2^DS^2^ score (0–10 points): Age ≥ 75 years (1 point), atrial fibrillation (1 point), dysphagia (2 points), male sex (1 point), stroke severity (NIHSS 0–4: 0 points, 5–15: 3 points, ≥ 16: 5 points).

Pneumonia Score (0–5 points): Age (< 65 years: 0 points, ≥ 65 years: 1 point), sex (female: 0 points, male: 1 point), NIHSS (0–10: 0 points, ≥ 11: 1 point), mechanical ventilation (no: 0 points, yes: 1 point), dysphagia (no: 0 points, yes: 1 point).

ISAN Score (0–21 points): mRS prestroke (Not Independent: 2 points), sex (female: 0 points, male: 1 point), age (< 60 years: 0 points, 60–69 years: 3 points, 70–79 years: 4 points, 80–89 years: 6 points, ≥ 90 years: 8 points), NIHSS (≤ 4: 0 points, 5–15: 4 points, 16–20: 8 points, ≥ 21: 10 points).

### Statistical analysis

Univariable analysis for SAP-related factors was performed using χ² test for categorical variables and Mann-Whitney U test for continuous variables. Group comparisons for categorical variables primarily used χ² test, with Fisher’s exact test (or Fisher-Freeman-Halton test when necessary) for cases with expected frequencies < 5.

Discriminative performance of each risk score was evaluated using ROC analysis. Area Under the Receiver Operating Characteristic Curve (AUROC) was calculated using nonparametric methods with 95% confidence intervals derived from asymptotic normal approximation based on nonparametric standard errors.

Pairwise comparison of ROC curves from the same cases was performed using nonparametric tests for AUC differences accounting for correlation in paired sample design. Optimal cutoffs were determined using Youden Index. For each cutoff, sensitivity, specificity, Positive Predictive Value (PPV), and Negative Predictive Value (NPV) were calculated along with Clinical Utility Index (CUI + = sensitivity × PPV) (CUI− = specificity × NPV).

Model calibration was assessed using calibration plots comparing predicted probabilities (grouped into quintiles) with observed proportions. Overall model performance was quantified using Brier score (calculated as the mean squared error for predicted probabilities).

Multivariable logistic regression analysis was performed to identify independent predictors of SAP. Variable selection was based on a pre-specified DAG constructed from literature review and clinical expertise. The minimal adjustment set included age, sex, NIHSS score, dysphagia, and mechanical ventilation. Atrial fibrillation was excluded due to sample size constraints (Events Per Variable; EPV = 4.4). Enteral feeding was excluded as a mediating variable to estimate the total effect of dysphagia on SAP.

#### Model assumption verification

Linearity of continuous variables (age, NIHSS) to logit was assessed using Box-Tidwell test, and multicollinearity was confirmed using Variance Inflation Factors (VIF) (all variables VIF < 2). Model calibration was assessed using Hosmer-Lemeshow test (χ²=6.69, df = 8, *p* =.571), and discrimination was evaluated using C-statistic (AUC) (AUC = 0.866). Due to limited event numbers, interaction terms were not included, and automatic variable selection was not used.

All statistical tests were two-sided with significance level set at *p* <.05. As analyses were primarily exploratory, no adjustment for multiple comparisons was performed. Statistical analyses were conducted using IBM SPSS Statistics version 30.0.

### Ethics statement

This study was conducted in accordance with the principles of the Declaration of Helsinki and received approval from the Ethics Committee of Ota Memorial Hospital (approval number: 341).

At our institution, patients or their legally authorized representatives routinely provide written informed consent upon admission, authorizing the use of de-identified clinical data for academic research purposes. Given the retrospective nature of this study and the use of data obtained during routine clinical practice, the Ethics Committee granted a waiver of additional study-specific informed consent in accordance with the Japanese Ethical Guidelines for Medical and Health Research Involving Human Subjects. Patient confidentiality and privacy were maintained through data anonymization.

The reporting of this study adhered to the STROBE (Strengthening the Reporting of Observational Studies in Epidemiology) guidelines.

## Results

### Patient flow and characteristics

**Fig. 1:**
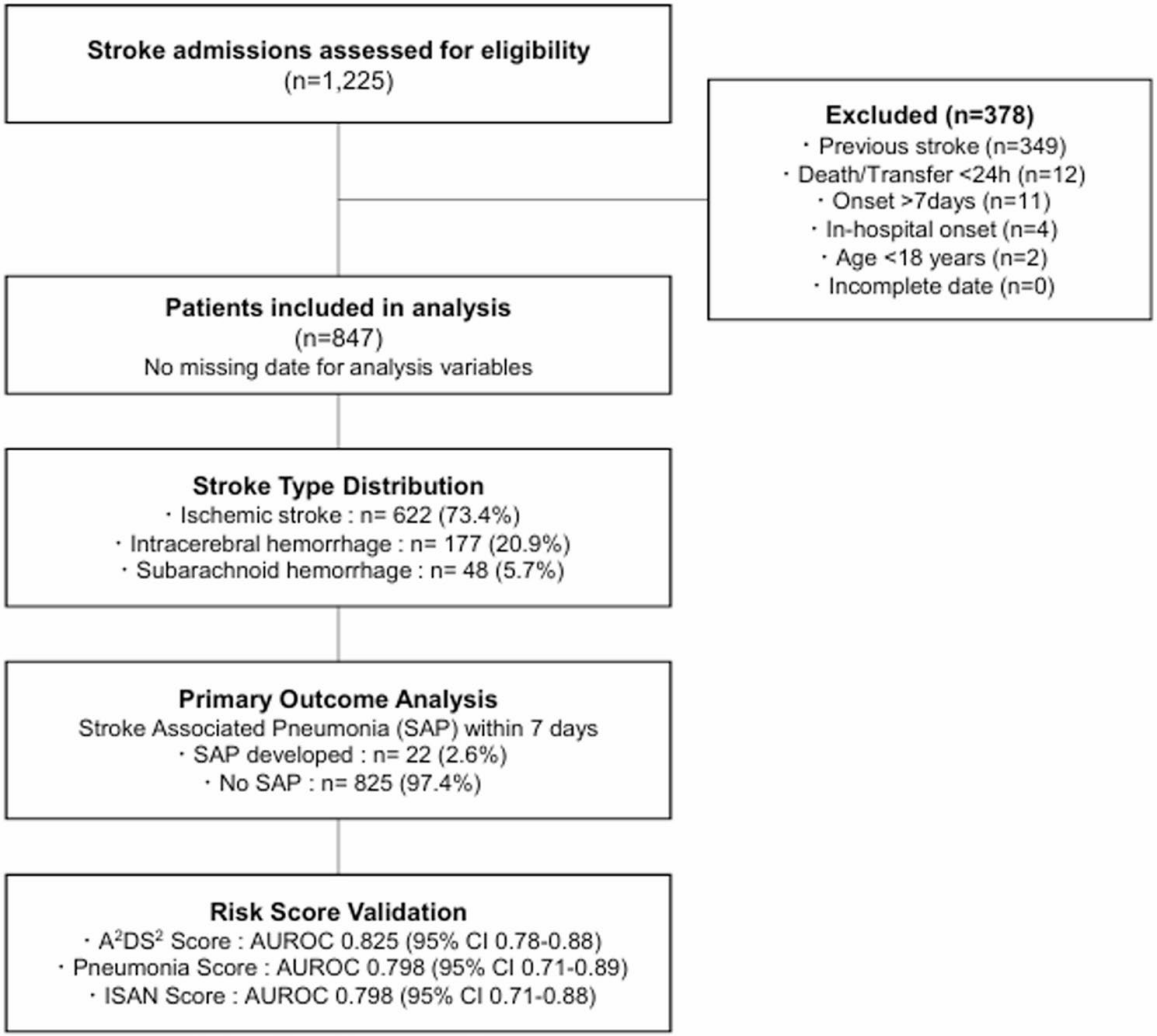
Study Flow Diagram

Of 1,225 stroke admissions during the study period, 378 were excluded: 349 with previous stroke, 12 with death or transfer within 24 h, 11 admitted beyond 7 days post-onset, 4 with in-hospital stroke, and 2 under 18 years. The final analysis included 847 cases with complete data for all analytical variables (Fig. 1).

The study cohort comprised 622 ischemic strokes (73.4%), 177 intracerebral hemorrhages (20.9%), and 48 subarachnoid hemorrhages (5.7%). Median age was 75 years (IQR 66–84), with 459 male patients (54.2%). Median NIHSS score was 4 (IQR 2–11), and 133 patients (15.7%) had pre-stroke disability (mRS ≥ 2).

### SAP incidence and timing

SAP developed in 22 patients (2.6%, 95% CI 1.5–3.7). SAP patients had significantly higher NIHSS scores (median 21 vs. 4, *p* <.001), more frequent dysphagia (90.9% vs. 48.1%, *p* <.001), greater need for enteral feeding (54.5% vs. 11.8%, *p* <.001), and more mechanical ventilation (18.2% vs. 1.5%, *p* <.001) compared to non-SAP patients. Length of stay was significantly prolonged in SAP patients (median 19 vs. 15 days, *p* =.030) (Table 1).

Temporal patterns showed early SAP predominance: median time to diagnosis was 2 days (IQR 1.00–3.25.00.25), with 17 cases (77.3%) occurring within 72 h and 5 cases (22.7%) on days 4–7.

**Table 1:** Baseline characteristics of the study population

	Total	SAP (+)	SAP (-)	*p*-value
	*n* = 847	*n* = 22	*n* = 825	
Men, n(%)	459(54.2)	15(68.2%)	444(53.8%)	*p* =.200
Age, median(IQR)	75(66–84)	82(70–90)	75(66–84)	*p* =.097
Stroke Type				
Ischemic stroke, n(%)	622(73.4)	13(59.1)	609(73.8)	*p* =.160
Intracerebral hemorrhage, n(%)	177(20.9)	8(36.4)	169(20.5)	
Subarachnoid hemorrhage, n(%)	48(5.7)	1(4.5)	47(5.7)	
Pre-Stroke mRS ≥ 2,n(%)	133(15.7)	4(18.2)	129(15.6)	*p* =.765
NIHSS Score, median(IQR)	4(2–11)	21(10–33)	4(2–10)	*p* <.001
Dysphagia, n(%)	417(49.2)	20(90.9)	397(48.1)	*p* <.001
Enteral feeding, n(%)	109(12.9)	12(54.5)	97(11.8)	*p* <.001
Mechanical ventilation, n(%)	16(1.9)	4(18.2)	12(1.5)	*p* <.001
Atrial fibrillation, n(%)	184(21.7)	8(36.4)	176(21.4)	*p* =.113
Hypertension, n(%)	734(86.7)	21(95.5)	713(86.4)	*p* =.342
Hyperlipidemia, n(%)	485(57.3)	11(50)	474(57.5)	*p* =.517
Diabetes mellitus, n(%)	261(30.8)	11(50)	250(30.3)	*p* =.061
History of alcohol, n(%)	105(12.4)	3(13.6)	102(12.4)	*p* =.747
Smoking history, n(%)	145(17.6)	3(15.8)	142(17.6)	*p* = 1.000
Length of stay, median(IQR)	15(11–21)	19(13–30)	15(11–21)	*p* =.030

*mRS* modified Rankin Scale, *NIHSS* National Institutes of Health Stroke Scale

### Stroke subtype analysis

SAP incidence varied by stroke subtype: ischemic stroke 2.1% (13/622), intracerebral hemorrhage 4.5% (8/177), subarachnoid hemorrhage 2.1% (1/48). Intracerebral hemorrhage showed numerically higher incidence, but the difference was not statistically significant (Fisher-Freeman-Halton exact test, *p* =.160).

### Risk score performance

All three risk scores demonstrated good discrimination for SAP prediction. A^2^DS^2^ score achieved the highest AUROC of 0.825 (95% CI 0.78–0.88), followed by Pneumonia Score (AUROC 0.798, 95% CI 0.71–0.89) and ISAN Score (AUROC 0.798, 95% CI 0.71–0.88). Pairwise comparisons showed no statistically significant differences: A^2^DS^2^ score vs. ISAN score (ΔAUC = 0.027, z = 0.956, *p* =.339), A^2^DS^2^ score vs. Pneumonia score (ΔAUC = 0.027, z = 0.750, *p* =.453), ISAN score vs. Pneumonia score (ΔAUC ≈ 0, z = 0.003, *p* =.998).

**Fig. 2:**
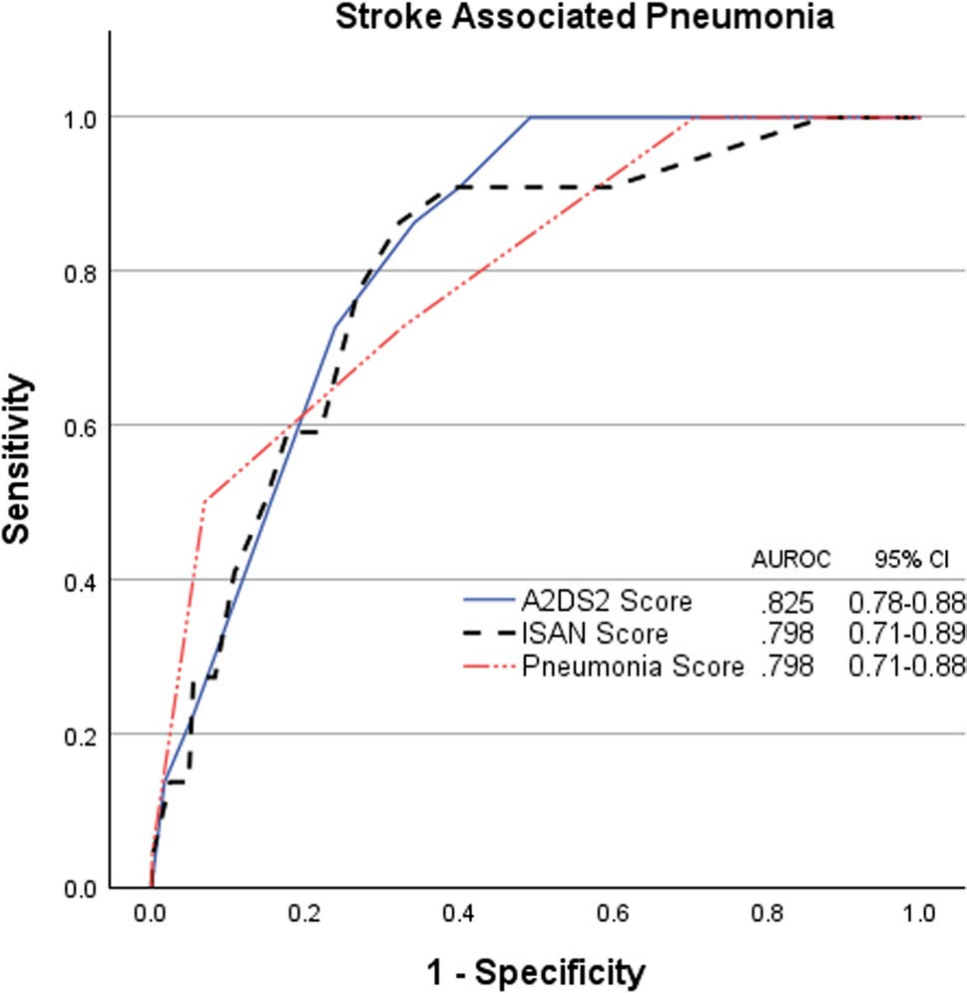
Receiver operating characteristic curves of Stroke Associated Pneumonia

Optimal cutoffs derived by Youden Index were: A^2^DS^2^ score ≥ 6 (sensitivity 86.4%, specificity 65.8%, PPV 6.3%, NPV 99.5%), Pneumonia Score ≥ 4 (sensitivity 50.0%, specificity 93.1%, PPV 16.2%, NPV 98.6%), ISAN Score ≥ 10 (sensitivity 86.4%, specificity 67.8%, PPV 6.7%, NPV 99.5%). Clinical Utility Index showed Pneumonia Score excelled in SAP exclusion (CUI- 0.917), while A^2^DS^2^ score provided balanced performance (Table 2; Fig. 2).

Sensitivity analysis using A^2^DS^2^ score cutoff ≥ 5 showed higher sensitivity (90.9%) but lower specificity (60.0%), with PPV 5.7% and NPV 99.6%. This lower threshold captured 20 of 22 SAP cases while maintaining excellent negative predictive value.

**Table 2:** Comparison of diagnostic performance and cutoff values of SAP prediction scores

	AUROC	95%CI	Youden Index	Cutoff	Sensitivity	Specificity	PPV	NPV	CUI+	CUI-	Brier
A^2^DS^2^ score	0.825	0.78–0.88	0.522	6	0.864	0.658	0.063	0.995	0.054	0.654	0.0243
			0.509	5	0.909	0.600	0.057	0.996	0.057	0.597	
Pneumonia score	0.798	0.71–0.89	0.431	4	0.500	0.931	0.162	0.986	0.081	0.917	0.0235
ISAN score	0.798	0.71–0.88	0.541	10	0.864	0.678	0.067	0.995	0.057	0.674	0.0245

*AUROC* Area Under the Receiver Operating Characteristic curve, *PPV* Positive Predictive Value, *NPV* Negative Predictive Value, *CUI*+ Clinical Utility Index Positive(Sensitivity × PPV), *CUI*- Clinical Utility Index Negative(Specificity × NPV)

### Model calibration

**Fig. 3:**
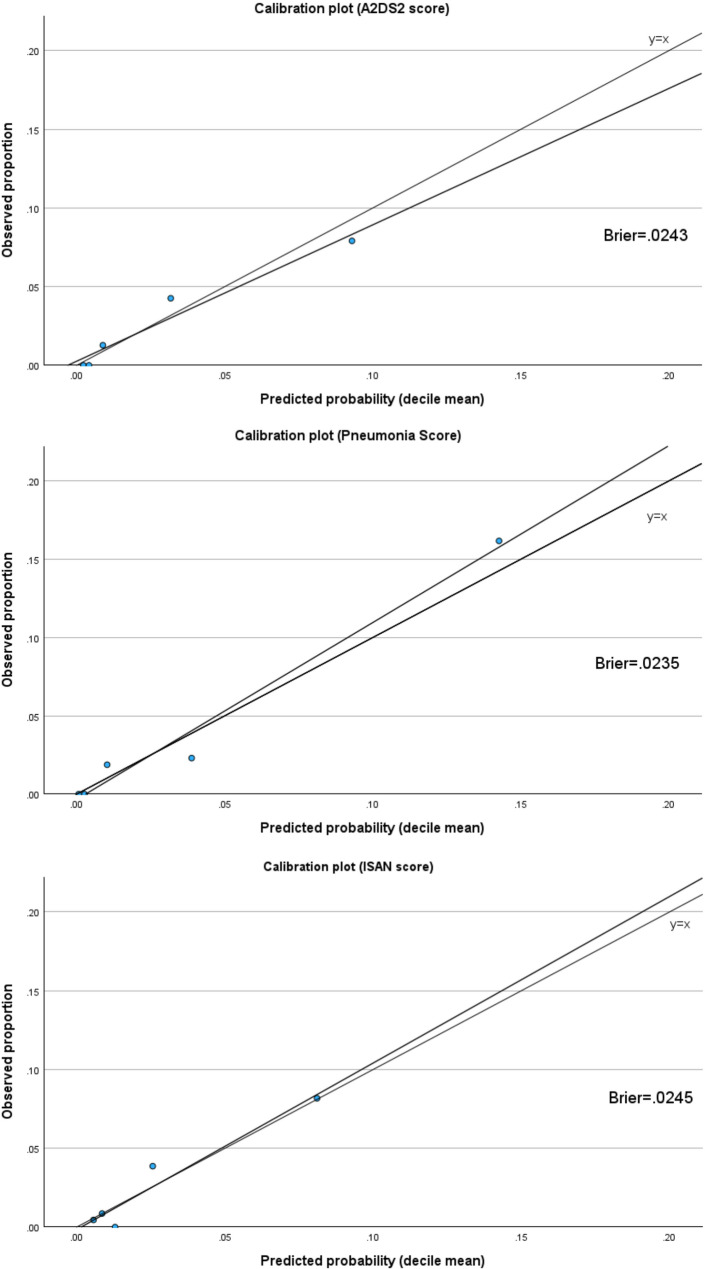
Comparison of calibration across SAP risk scores

Calibration assessment showed reasonable agreement between predicted and observed probabilities for all scores. Calibration plots demonstrated good calibration in the low-risk range with slight overestimation in the high-risk range. Brier scores were similar across models (A^2^DS^2^ score 0.0243, Pneumonia score 0.0235, ISAN score 0.0245) (Fig. 3).

### Independent predictors of SAP

Multivariable logistic regression analysis identified four independent predictors of SAP (Table 3):

Male sex: OR 3.87 (95% CI 1.41–10.61, *p* =.009).

NIHSS score: OR 1.08 per point increase (95% CI 1.03–1.12, *p* <.001).

Dysphagia: OR 5.31 (95% CI 1.16–24.34, *p* =.031).

Mechanical ventilation: OR 5.33 (95% CI 1.28–22.18, *p* =.021).

Age showed a trend toward significance (OR 1.04 per year, 95% CI 1.00–1.08, *p* =.084). The model demonstrated good discrimination (C-statistic 0.866) with no evidence of multicollinearity (all VIF < 2.0).

**Table 3:** Multivariable analysis of risk factors for SAP

	OR	95%CI	*p*-value
Men	3.87	1.41–10.61	*p* =.009
Age	1.04	1.00–1.08	*p* =.084
NIHSS Score	1.08	1.03–1.12	*p* <.001
Dysphagia	5.31	1.16–24.34	*p* =.031
Mechanical ventilation	5.33	1.28–22.18	*p* =.021

*NIHSS* National Institutes of Health Stroke Scale

## Discussion

### Principal findings and international comparison

In this study, we observed a SAP incidence of 2.6% (95% CI 1.5–3.7) among 847 stroke patients in a Japanese acute care hospital and evaluated the diagnostic performance of three risk prediction scores. This incidence rate is positioned at the lower end of the internationally reported range of 2.3% to 44% [2].

When interpreting these differences in incidence rates, multiple factors must be considered. Our cohort (median age 75 years, 54.2% male, median NIHSS 4) had similar patient characteristics to the German study by Hoffmann et al. [5] (age 72 years, 50.6% male, NIHSS 4, SAP 7.2%) and the UK study by Smith et al. [7] (age 76 years, 53% male, NIHSS 4, SAP 6.7%). However, the lower SAP incidence in our study suggests the involvement of factors beyond patient characteristics.

An analysis of 413,000 cases from England and Wales demonstrated that SAP incidence varied from 0.82% to 21.4% between stroke units, with patient clinical characteristics explaining only 5% of the observed variation [16]. This finding highlights the importance of care processes and organizational structures. In our hospital, the following are implemented: nurse-to-patient ratios of 1:3 in the SCU and 1:10 in general wards, systematic swallowing evaluation by a multidisciplinary team, early aspiration prevention protocols, and frequent patient monitoring. These organizational approaches may be associated with SAP prevention.

Additionally, differences in diagnostic criteria should be considered. While this study employed modified CDC criteria requiring new infiltrates on chest X-ray as a mandatory criterion, other studies have used criteria that do not mandate radiological confirmation or definitions based solely on antibiotic administration [17]. These methodological differences may influence the reported incidence rates.

### Characteristics of SAP onset timing and the importance of early intervention

In this study, 77.3% of SAP cases occurred within 72 h. de Jonge et al. (2022) reported that 64% occurred within the first 7 days with a peak on day 3 [18], indicating that our study demonstrated an even more concentrated pattern in the early period. Following stroke, Stroke-Induced Immunosuppression Syndrome (SIIS) occurs through activation of the Hypothalamic-Pituitary-Adrenal (HPA) axis and sympathetic nervous system, which, along with clinical factors such as dysphagia, becomes a major risk factor for post-stroke infection [19]. These pathophysiological changes increase the risk of SAP development in the acute phase.

The absence of statistically significant differences in patient characteristics between the early-onset and late-onset groups represents an important finding. This suggests that the timing of SAP onset may be influenced not only by individual patient factors but also by other factors such as care processes during the acute phase. Bray et al. (2017) reported that delays in swallowing screening and comprehensive swallowing assessment were associated with increased SAP risk, with comprehensive swallowing assessment particularly showing a dose-response relationship whereby each day of delay was associated with a 1% absolute increase in SAP incidence [20]. These findings support the potential critical role of early care processes in SAP prevention.

### Diagnostic performance of risk scores

All three risk scores (A^2^DS^2^, Pneumonia Score, ISAN) demonstrated good discriminative ability (AUROC 0.798–0.825), with no statistically significant performance differences observed between them. The AUROC of 0.825 for the A^2^DS^2^ score was comparable to the 0.835 reported in the original development study by Hoffmann et al. (2012) [5], confirming external validity in the Japanese population.

The negative predictive value of 99.5% at the optimal cutoff of ≥ 6 has important clinical implications. This high negative predictive value enables reliable identification of low-risk patients, allowing for efficient allocation of limited healthcare resources. Alternatively, when prioritizing sensitivity, a cutoff of ≥ 5 (sensitivity 90.9%, negative predictive value 99.6%) may be considered.

However, caution is warranted regarding the generalizability of our findings. In a systematic review by Kishore et al. (2016), nine SAP prediction scores were evaluated, showing overall similar discriminative ability and calibration, yet demonstrating variability in performance across external validation cohorts (C-statistics 0.67–0.83) [12]. This variability may reflect differences in patient population characteristics and SAP diagnostic criteria. The optimal cutoff values identified in this study also require prospective validation in other healthcare settings and different patient populations.

### Pathophysiological considerations of independent risk factors

Dysphagia (OR 5.31, 95% CI 1.16–24.34) was identified as a significant risk factor in this study. In a meta-analysis by Li et al. (2024), the OR for dysphagia was reported as 3.10 (95% CI 2.26–4.27), which is generally consistent with our findings. However, the wide 95% confidence interval in our study should be noted as a limitation in the precision of the estimate [21].

The finding that male sex was an independent risk factor is also consistent with previous cohort studies [21]. While reviews on immunological mechanisms of sex differences exist, they primarily focus on viral respiratory infections and do not provide direct evidence for SAP [22]. Additionally, social factors such as higher smoking rates among men compared to women may contribute to this association [23].

Regarding the association with mechanical ventilation (OR 5.33, 95% CI 1.28–22.18), multiple mechanisms are implicated. Zuercher et al. (2019) reported that laryngeal and pharyngeal mucosal injury from endotracheal tubes, restriction of laryngeal elevation by cuffed tubes, suppression of swallowing reflexes by sedatives, and ICU (Intensive Care Unit) -acquired weakness contribute to post-extubation dysphagia [24]. In stroke patients, these mechanisms superimposed on neurological deficits are thought to further increase the risk of dysphagia and aspiration pneumonia.

Higher NIHSS scores (OR 1.08 per point, 95% CI 1.03–1.12) also represent an important risk factor, reflecting stroke severity. Although age (OR 1.04 per year, 95% CI 1.00–1.08, *p* =.084) did not reach statistical significance, it showed a clinically meaningful trend, likely reflecting systemic frailty. In a meta-analysis by Ahmad et al. (2024), both higher NIHSS scores and advanced age were reported as significant predictors of pneumonia development [25], which is generally consistent with our findings.

### Study limitations and future perspectives

This study has several important limitations.

First, as a single-center study, there are inherent restrictions on the external validity and generalizability of the results. The characteristics of our hospital (nurse-to-patient ratios of 1:3 in the SCU and 1:10 in general wards, 24-bed stroke care unit, systematic swallowing screening protocols, regular interventions by speech-language pathologists and dental hygienists) may not represent all Japanese hospitals or international settings. These factors likely contribute to the observed low SAP incidence, limiting the applicability of our findings to facilities with different resource levels or care models.

Second, the retrospective design precluded systematic collection of several important variables. Data on concomitant medications that may influence stroke or SAP risk (including antipsychotics, antineoplastic agents, and immunosuppressants) were lacking. Additionally, the presence of hereditary vascular diseases (CADASIL, fibromuscular dysplasia, etc.) could not be assessed, which, although rare, may affect both stroke risk and dysphagia. Detailed smoking history beyond current smoking status, pre-stroke swallowing function, nutritional status indicators, oral hygiene markers, and specific preventive intervention protocols were not captured.

Third, with only 22 SAP cases, the multivariable logistic regression analysis yielded only 4.4 events per variable. This constraint necessitated DAG-based variable selection, requiring exclusion of potentially important factors such as atrial fibrillation from the multivariable model. The obtained odds ratios have wide confidence intervals and should be considered exploratory, requiring validation in larger cohorts before clinical implementation.

Fourth, the heterogeneity in swallowing assessment represents a significant methodological limitation. Two different assessment tools (Fujishima Grade and MASA) were used without standardized selection criteria or documented decision-making processes. Inter-rater reliability was not assessed, potentially introducing measurement bias that could affect the reported association between dysphagia and SAP.

Fifth, the strict application of modified CDC criteria requiring radiological confirmation may have underestimated SAP incidence compared to studies using clinical diagnosis alone. However, this approach enhanced diagnostic specificity and reduced false-positive classifications.

Future research should address these limitations through several approaches. Multicenter prospective studies incorporating diverse Japanese hospitals could establish more generalizable incidence estimates. Collaboration with national registries such as the Japan Stroke Data Bank and international cohorts (UK Sentinel Stroke National Audit Programme [SSNAP]; US Get With The Guidelines-Stroke) would enable robust benchmarking. Development of machine learning models using larger datasets may better handle rare event data and identify complex interaction patterns.

## Conclusion

In this study of 847 stroke patients (median age 75 years, median NIHSS 4, ischemic stroke 73.4%) in a Japanese acute care hospital, we identified a SAP incidence of 2.6% (95% CI 1.5–3.7). This incidence rate reflects the specific characteristics of our patient population, stringent diagnostic criteria (mandatory radiological confirmation using modified CDC criteria), and our hospital’s healthcare delivery system.

The A^2^DS^2^ score demonstrated excellent discriminative ability (AUROC 0.825, 95% CI 0.78–0.88) and high negative predictive value (99.5% at cutoff ≥ 6), enabling reliable identification of low-risk patients. Male sex (OR 3.87, 95% CI 1.41–10.61), stroke severity (NIHSS OR 1.08 per point, 95% CI 1.03–1.12), dysphagia (OR 5.31, 95% CI 1.16–24.34), and mechanical ventilation (OR 5.33, 95% CI 1.28–22.18) were identified as independent predictors of SAP. The observation that 77.3% of SAP cases occurred within 72 h post-onset suggests the importance of intensive monitoring of high-risk patients during this period.

Importantly, the SAP incidence and prediction score performance observed in this study may reflect our hospital’s specific environment and patient population, requiring caution when directly applying these findings to other healthcare settings. The wide confidence intervals of the obtained odds ratios indicate that these results should be interpreted as exploratory. As a single-center study, generalization of our findings requires careful consideration.

Future multicenter collaborative studies incorporating diverse patient populations and healthcare settings are needed to validate these results and establish risk stratification-based SAP prevention strategies optimized for Japanese healthcare environments. Particularly, validation with larger sample sizes is expected to enable the development of more reliable risk prediction models and implementation of cost-effective prevention protocols.

## Supplementary Information

Supplementary Material 1.

## Data Availability

The datasets generated and/or analyzed during the current study are not publicly available due to institutional and ethical restrictions, but are available from the corresponding author on reasonable request.
