# Supplementary figures and images for: Stroke-associated pneumonia in Japanese acute care settings: incidence and preliminary validation of risk prediction scores
Source: BMC Neurol. 2025 Dec 11;25:499. doi: 10.1186/s12883-025-04523-8 (PMC12699884; doi:10.1186/s12883-025-04523-8)

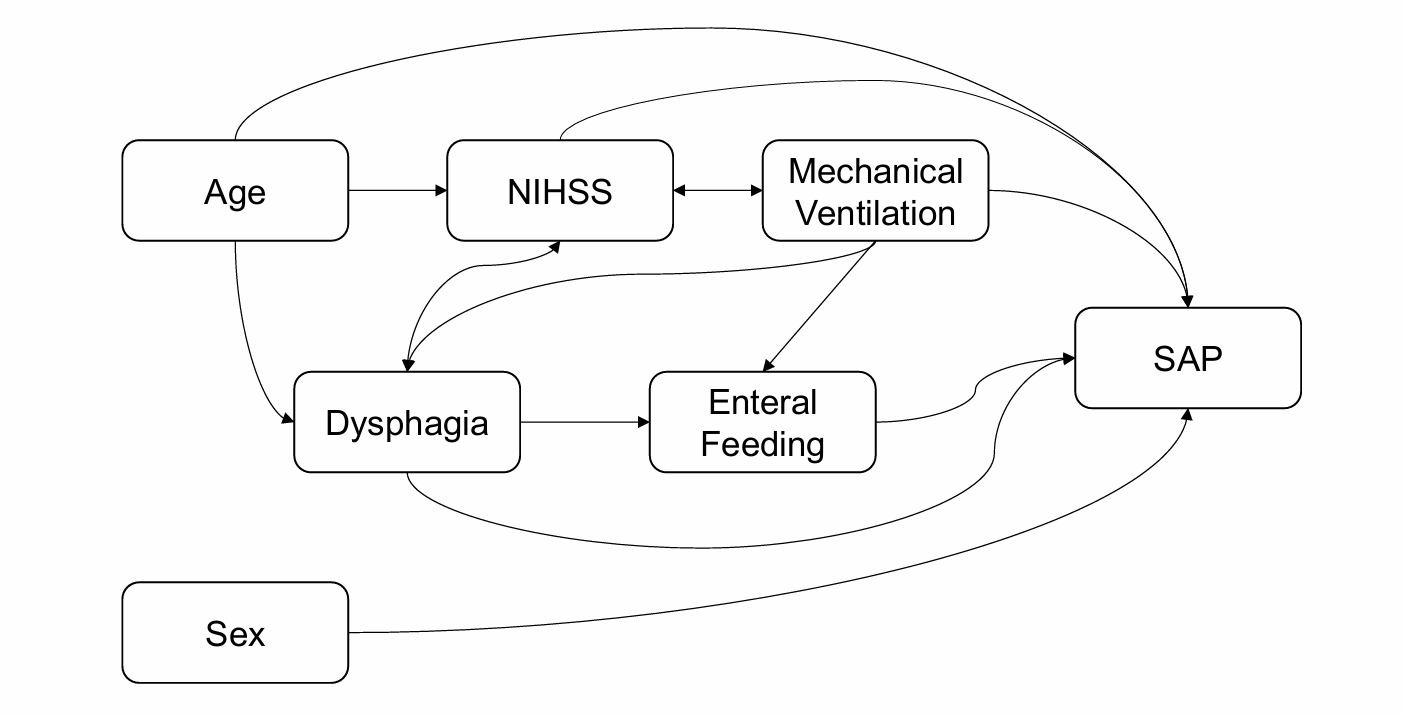


Figure S1. Directed Acyclic Graph

Supplement: Supplementary file 1 — Supplementary Material 1. [file 12883_2025_4523_MOESM1_ESM.docx]
